# Dickkopf 3 as a New Monitoring Tool for Kidney Function After Living Kidney Donation

**DOI:** 10.3390/jcm13237454

**Published:** 2024-12-07

**Authors:** Antonia Schuster, Louisa Steines, Bernhard Banas, Tobias Bergler

**Affiliations:** 1 Department of Nephrology, University Hospital Regensburg, Franz-Josef-Strauß-Allee 11, 93053 Regensburg, Germanybernhard.banas@ukr.de (B.B.); tobias.bergler@klinikum-ingolstadt.de (T.B.); 2 Medical Clinic III-Nephrology, Hospital Ingolstadt, Krumenauerstr. 25, 85049 Ingolstadt, Germany

**Keywords:** DKK3, non-invasive monitoring, living kidney transplantation

## Abstract

**Background:** Even today, a non-invasive biomarker to identify donors with enhanced risk for renal impairment is missing. Dickkopf 3 (DKK3) can cause tubulointerstitial fibrosis and is associated with rapid eGFR loss. The aim of our work was to analyze whether DKK3 can be used as a non-invasive alert marker for an increased risk of loss of kidney function in living kidney donors (LKDs). **Methods:** All donors who were examined between July 2022 and June 2023 (*n* = 117) were included. DKK3 was measured in the urine. The collected patient-related data were compared with parameters before donation. The study cohort was stratified by DKK3 values (</≥200). **Results:** In the follow-up, 89 donors had a DKK3 value < 200 (group 1) and 28 donors had a DKK3 value ≥200 (group 2). During post-donation follow-up, renal function in group 1 was significantly better than that in group 2 (*p* = 0.01), although no difference in renal function before donation was detected (*p* = 0.84). Group 2 showed also a greater eGFR loss over time than group 1. **Conclusions:** LKDs with elevated DKK3 levels in the FU had impaired kidney function without evidence of increased risk factors pre-donation. DKK3 can represent a possible monitoring tool for kidney function in LKDs.

## 1. Introduction

Due to stagnating numbers of deceased kidney donations, living kidney donation is playing an increasingly important role in Germany [1]. In addition to a careful donor selection, consistent follow-up of donors is an important medical task to minimize the lifetime risk of developing chronic kidney disease. Hori et al. showed in their work that regular and long-term follow-up care of living kidney donors is important to maintain kidney function, as complications that affect long-term survival often only occur more than 10 years after donation [2].

According to the KDIGO guidelines, creatinine, eGFR and albuminuria should be monitored regularly [3]. However, these parameters only reflect the current state of renal function. A non-invasive, easy-to-use biomarker for identifying donors with an increased risk for an intensified loss of function is not available.

The KDIGO guidelines also recommend that donors with chronic kidney disease should be treated in the same way as patients with chronic kidney disease due to other causes [3]. Dickkopf 3 (DKK3), a profibrotic glycoprotein, has recently been identified as a biomarker for estimating the risk of rapid eGFR loss in patients with chronic kidney disease. Zewinger et al. were able to show that patients with elevated DKK3 levels in the urine and a known chronic kidney disease regardless of the cause showed more tubulointerstitial fibrosis and a rapid loss of eGFR [4]. As a result of these investigations, DKK3 is now being used more and more in everyday clinical practice.

The proteins of the Dickkopf family (DKK1-4, DKK-like protein 1), to which DKK3 belongs, influence the Wnt signaling pathway. The Wnt signaling pathway plays an important role in embryogenesis as well as in the development of various diseases, such as in the development of ADPKD in the context of the kidney [5,6]. DKK3 has not been detected in healthy adult cells. In various animal models, however, it has been shown that DKK3 is secreted by stressed tubular epithelial cells after kidney damage, is detectable in the urine and triggers a profibrotic T cell response [7].

In an earlier study from our working group, we were able to show, in a cohort of 122 kidney transplant patients, that DKK3 correlates with kidney function and can also forecast kidney function over a period of three years [8]. Recently, de Fallois et al. were able to show in their study on kidney transplant patients that the urine DKK3 value of their donors shortly before donation, both in the context of deceased donation and living donation, represents the subsequent kidney function of the recipients. The uDKK3/crea donor level was identified as an independent predictor of subsequent kidney function. Kidney recipients with uDKK/crea levels below the median had better eGFR 3 months after transplantation than the comparison group with values above the median [9].

Therefore, DKK3 is also a possible biomarker for estimating the risk of kidney function deterioration in patients after kidney transplantation.

The aim of our current work was to analyze DKK3 in the urine of patients after living kidney donation. For this purpose, DKK3 was determined from all living kidney donors, which are followed up annually in our outpatient clinic. Patient-related data as well as laboratory parameters were also included in the analysis. We conducted two different analyses: (1) comparison of resulting eGFR based on DKK3 stratification; (2) evaluation of eGFR at distinct time points after LKD based on DKK3 levels. In addition, the 14-day biopsies of the corresponding kidney transplant recipients were analyzed with regard to chronic changes in the sense of interstitial fibrosis and tubular atrophy (IFTA), which can be viewed as previous damage coming from the donor in this early phase after transplantation. Patients were then stratified according to IFTA < or ≥5% and compared with the DKK3 values.

The aim of this study was to determine whether DKK3 can be used in the follow-up care of kidney donors as a possible non-invasive alert marker for an enhanced risk of renal impairment.

## 2. Material and Methods

### 2.1. Patients’ Baseline Characteristics

All living donors, who were followed up in our transplant clinic once a year, were included in the study (*n* = 117). Patients were recruited between 1 July 2022 and 30 June 2023. As part of the appointment, a urine sample was collected and DKK3 was determined in these samples. The patient-related data and laboratory parameters routinely gathered from the patients during their presentation were analyzed. In addition, the laboratory and examination findings of the patients assembled as part of the preparation for their living kidney donation were included. The study cohort was then classified according to DKK3 values (</≥200). These cut-off values were chosen based on the recommendation of the examining laboratory. If the DKK3 value was less than 200 pg/mg crea, the result was seen as negative. This study was approved by the Ethical Committee of the University of Regensburg (22-2833-101).

### 2.2. Dickkopf 3 Analysis

The urine samples were determined in an external laboratory using a commercially available ELISA according to the manufacturers’ recommendations (DiaRen, Homburg, Germany).

### 2.3. Histological Analysis

The 14-day biopsies of the kidney recipients were analyzed for the presence of interstitial fibrosis and tubular atrophy. In this early phase after kidney donation, these chronic changes can be attributed to the donors. In total, 51 biopsies were identified. The remaining patients either did not receive a biopsy or the results of the biopsies were not available. The biopsies were then stratified based on the presence of IFTA < or ≥ 5% and correlated with the DKK3 values of the corresponding kidney donors.

### 2.4. Statistical Analysis

Continuous variables are shown as mean ± standard deviation and categorical data are presented as frequency distributions (*n*) and percentages (%). Statistical analyses were carried out by a Student *t*-test, with a *p*-value < 0.05 indicating statistical significance.

## 3. Results

### 3.1. Stratification According to DKK3 Values </≥ 200 pg/mg Crea

**Figure 1:**
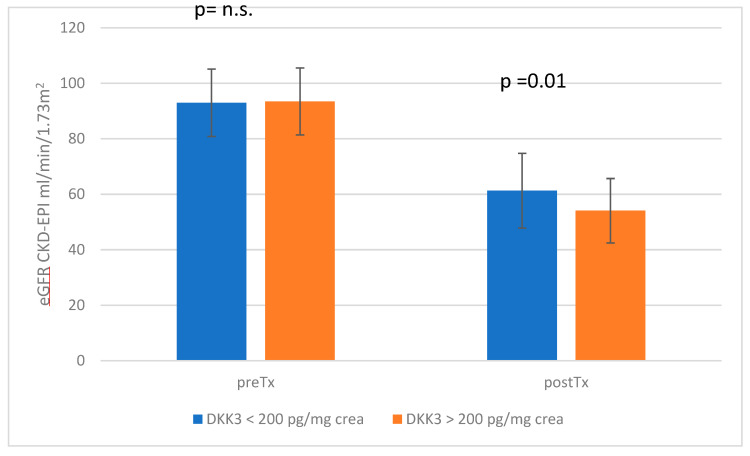
eGFR comparison of living kidney donors with DKK3 values < and ≥ 200 pg/mg crea before and after donation. N.s. = not significant.

In our cohort of 117 donors, a total of 89 donors, of which 31 were men and 58 were women, had a DKK3 value of <200 (group 1) and 28 donors, 14 men and 14 women, had a value ≥ 200 (group 2). At the time of presentation, group 1 had an average of 8.7 ± 4.9 years after living donation, whereas group 2 had donated 6.7 ± 5.2 years ago. The mean creatinine value was 1.12 ± 0.26 mg/dL for group 1 and 1.23 ± 0.25 mg/dL for group 2 (*p* = 0.05); the corresponding eGFR (CKD-EPI) was 61.27 ± 13.47 mL/min in group 1 and 54.07 ± 11.64 mL/min in group 2 (*p* = 0.01). The eGFR course of both cohorts is shown graphically in Figure 1. Albuminuria was 18.02 ± 52.1 mg/g crea in group 1 and 12.27 ± 20.9 mg/g crea in group 2 (*p* = 0.57). Arterial hypertension was present in a similar number of patients, without statistical significance (*p* = 0.8).

The mean DKK3 values of the donors with a value of less than 200 were 39.06 ± 30.28 pg/mg crea, whereas donors stratified in the intensified DKK3 group had mean values of 2185.11 ± 2153.69 pg/mg crea (*p* = 4.1 × 10^−16^).

Prior to kidney donation, both groups showed no statistically significant difference in renal function (group 1: creatinine 0.78 ± 0.14 mg/dL, eGFR 92.95 ± 12.17 mL/min; group 2: creatinine 0.77 ± 0.11 mg/dL, eGFR 93.46 ± 12.1 mL/min; creatinine *p* = 0.72, eGFR *p* = 0.84). BMI (*p* = 0.34) and blood pressure values did also not differ significantly between both groups (group 1: 122/76 mmHg; group 2: 122/77 mmHg; *p* = 0.89 (systolic RR); and *p* = 0.44 (diastolic RR)). The other parameters recorded before kidney donation (e.g., kidney scintigraphy findings, etc.) also showed no relevant difference between the two groups. The baseline data of both cohorts are shown in Table 1.

In a further analysis, the eGFR loss of each individual patient between the current presentation and the time before kidney donation was calculated. This individual eGFR loss was then adjusted to the time after transplantation so that the patient’s annual eGFR could be determined. The individual eGFR loss of all patients in group 1 was then compared with the eGFR loss of all patients in group 2. Through this, the average overall eGFR loss in the low-DKK3 group was 32.1 ± 10.25 mL/min and the average loss in the high-DKK3 group was 39.24 ± 15.15 mL/min (*p* = 0.006). If we put the eGFR loss in relation to the time after kidney donation, the result is a mean eGFR loss of 6.44 ± 7.7. mL/min for the low-DKK3 group and of 13.3 ± 13.9 mL/min in the high group (*p* = 0.0015).

As the eGFR slope is not a linear function, we additionally examined the eGFR at distinct time points in patients with a DKK3 value < 200 and a DKK3 value > 200. The examined time points were 2 years, 5 years, 10 years and 15 years after donation. This analysis showed that the group with DKK3 values < 200 had a better eGFR than donors with DKK3 values > 200.

**Figure 2:**
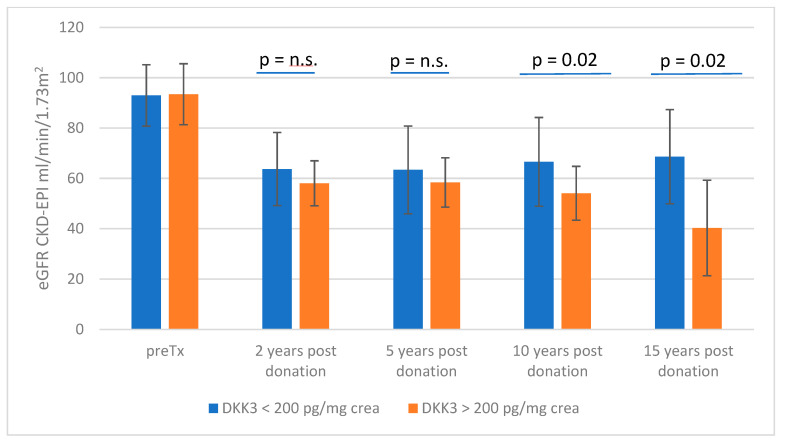
The eGFR course at distinct time points stratified by DKK > and < 200 pg/mg crea. N.s. = not significant.

At 2 years after donation, the mean eGFR was 63.73 ± 14.6 mL/min in group 1 (*n* = 79) and 58.08 ± 8.9 mL/min in group 2 (*n* = 26). The statistical significance level is not clearly reached here, but a clear trend can be seen (*p* = 0.06). This trend was also confirmed at the 5-year time point (group 1: eGFR 63.4 ± 17.5 mL/min, *n* = 75; group 2: eGFR 58.41 ± 17.0 mL/min, *n* = 17; *p* = 0.26). After 10 years, a statistically significant difference is demonstrated (group 1: eGFR 66.6 ± 17.6 mL/min, *n* = 45; group 2: eGFR 54.09 ± 10.7 mL/min, *n* = 11; *p* = 0.02), which persists even after 15 years (group 1: eGFR 68.65 ± 18.6 mL/min *n* = 17; group 2: eGFR 40.3 ± 19.0 mL/min, *n* = 3; *p* = 0.02). In addition to the differences in eGFR, it is also noticeable that group 2 shows a significantly greater decrease in eGFR over time than group 1, where an almost constant course in eGFR can be proven. The eGFR course can be seen in Figure 2.

### 3.2. Results of the Biopsy Analysis

Of the 117 donors, 51 of the corresponding recipients had a 14-day biopsy with information about the presence of IFTA. Of these, 31 kidney donors had IFTA values less than 5%, whereas 20 patients had more than 5% IFTA noted. One patient had 60% IFTA, which was the highest recorded value. If we now compare the measured DKK3 values at the time of presentation between these groups, there is no statistically significant difference between the two groups (*p* = 0.26). The patients with little IFTA had a DKK3 value of 853 pg/mg crea ± SD, whereas the donors with more IFTA had a DKK3 value of 637 pg/mg crea ± SD. However, the one patient with 60% IFTA showed a significantly increased DKK3 value of 3523 pg/mg crea.

## 4. Discussion

In our study, we examined DKK3 in the urine of living kidney donors with a meaningful time of follow-up. We were able to show that kidney donors with elevated DKK3 values (>200 pg/mg crea) showed worse kidney function over the years. Differences in risk factors such as arterial hypertension or diabetes mellitus could not be traced between both groups.

There is a discussion in the literature of if the chronic damage (IFTA) found in early biopsies (≤14 days) of the recipients allows one to drawn a conclusion about the organ quality and organ function of the corresponding living donors.

Hori et al. analyzed the presence of IFTA in 1 h allograft biopsies from 116 living donors in their study. Their observations suggest that the presence of IFTA has an influence and is a possible predictor of the remnant renal function of kidney donors [10]. In contrast, Emmons et al. could not detect a difference in the kidney function of the kidney donors depending on the histological findings in an implantation biopsy. Chronic changes were detected in 14% of the donors, with vascular changes predominating. However, kidney function after 6 months showed no difference [11]. Buus et al. provided similar results in their work on 49 living donors and 51 recipients. They analyzed both the cortex volume of both donor kidneys by contrast-enhanced computed tomography and histomorphometric parameters. The cortex volume but not the histological changes could be identified as a predictor of the 1-year kidney function of the donors. In contrast, IFTA was a prognostic parameter for the function of the kidney recipients [12]. Looking at our analysis in comparison to the observations mentioned, it should be noted that we saw no difference in the frequency of IFTA, but the group with DKK3 values > 200 showed a worsened eGFR over time. The results of the biopsy, therefore, do not allow any conclusions to be drawn about the course of kidney function after the donation.

As we saw no difference in the occurrence of IFTA between the two groups, the elevated DKK3 levels in the follow-up did not appear to be due to fibrosis at the time of donation. Nevertheless, it should be noted here that the small number of cases is a possible explanation that no difference is detectable. Only the patient with IFTA values above 50% showed highly elevated DKK3 values.

Janki et al. compared 761 living kidney donors with non-donors in terms of kidney function. The eGFR of the donors was 27.23 mL/min lower than that of the non-donors after 8 years of follow-up. Looking at the eGFR of the donors alone, there was a decrease in eGFR from baseline to the end of follow-up of 32.7 mL/min [13].

In a review by Li et al., it was shown that the greatest eGFR loss (−18.64 mL/min) occurs in the first 6 months and not over the course of the following years. After ten years and longer, this study showed an eGFR loss of −17.84 mL/min [14]. Hori et al. recognized the greatest eGFR loss in the first 3 months after donation and were then able to observe a stabilization in kidney function in their cohort [2].

These studies are, therefore, comparable to our results. When looking at the individual eGFR progression of the patients, Li et al. saw the greatest eGFR loss in the first year, which also applies to some of our patients. The group with DKK3 > 200 pg/mg crea showed a statistically significant higher eGFR loss over time. Even taking into account the difference in the number of cases, there is a difference in the eGFR progression between the two groups, particularly at later time points. It is worth mentioning that patients with DKK3 values < 200 had stable and improved eGFR values in comparison to patients with DKK3 values > 200 even after 10 and 15 years. DKK3, therefore, appears to indicate a change in kidney function in the long term, too.

This observation is also consistent with previous studies in other cohorts. Zewinger et al. showed in their work on patients with chronic kidney disease that increased DKK3 levels were associated with worsened kidney function [4]. This finding was then confirmed in our own analysis of patients after a kidney transplantation, where increased DKK3 levels were associated with impaired graft function and where DKK3 could also predict kidney function [8]. An observation that could also be made by de Fallois et al. In their work, the DKK3 value of the donors before transplantation was an independent predictor of the subsequent kidney function and the organ quality after transplantation [9].

In contrast, Jehn et al. published recently a study in which DKK3 was compared before and shortly after living kidney donation and one year later. Here, no clear prognostic influence of DKK3 on kidney function could be found. As in the above-mentioned work, the living kidney donors showed the greatest loss of eGFR in the first period after nephrectomy and kidney function stabilized at a better level in the course of the first year. The Dickkopf 3 values were highest in the first days after the donation and were still elevated after one year, although slightly decreasing. However, the living kidney donors showed increased DKK3 values one year after donation, but a subsequent deterioration in kidney function could not be detected in the following two years [15]. Thus, these results show that even in living kidney donors without a recognizable pathology of the kidneys, a dynamic and an increase in the Dickkopf 3 values above the reference range can be found, which was also evident in some of our patients.

There is currently no existing biomarker to determine whether a candidate is suitable as a kidney donor. As a possible assisting tool, the “ESRD Risk Tool for Kidney Donor Candidates”—Calculator was developed. This tool allows one to calculate both the 15-year and lifetime risk of end-stage kidney disease without donation based on the various patient-related data and demographic values included. This value must then be combined with the risk that arises from a donation [16]. The currently available results, primarily due to the monocentric and retrospective nature of our analysis, certainly do not allow one to prohibit a donation due to increased DKK3 values. A possible approach could be to consider the DKK3 values together with the results of the calculator as part of the evaluation of a living kidney donor. If the preparation shows significantly increased DKK3 values and the indicated ESRD risk is noticeable, which both cannot be explained plausibly, carrying out an additional workup, e.g., by kidney biopsy, could be taken into consideration. Since there was no marked deterioration in kidney function, i.e., ESRD, in our two cohorts, the pre-donation eGFR values were retrospectively suitable for donation. Since the DKK3 values before kidney donation are not available in our analysis, further studies in larger cohorts are certainly necessary to substantiate the significance of DKK3 before donation.

In addition to the careful selection of a donor, regular and reliable follow-up care is also mandatory in the care of living kidney donors. However, there are currently no sufficient parameters to identify patients with an increased risk of impaired kidney function in the follow-up. DKK3 could, therefore, be included in the follow-up care of living kidney donors. For example, the patients’ DKK3 values after one and three years could be determined, and one could subsequently monitor and treat patients with elevated values (e.g., DKK3 values > 200) for existing risk factors for a deterioration in kidney function (blood pressure, HbA1c, etc.) as well as provide a more intensive follow-up.

## Figures and Tables

**Table 1:** Baseline data of both cohorts.

	Dickkopf 3 < 200 pg/mL	Dickkopf 3 > 200 pg/mL	*p*-Value
creatinine (mg/dL)	0.78	0.77	0.72
eGFR (mL/min)	92.95	93.46	0.84
creatinine clearance (ml/min)	132.04	126.93	0.56
body mass index (kg/m^2^)	25.73	26.40	0.35
HbA1c (%)	5.49	5.45	0.66
systolic blood pressure—day (mmHg)	125	126	0.67
diastolic blood pressure—day (mmHg)	79	80	0.64
systolic blood pressure—night (mmHg)	111	112	0.95
diastolic blood pressure—night (mmHg)	69	69	0.97

## Data Availability

If requested, original data subsets can be provided by the corresponding author.
